# Analysis of a model for surfactant transport around a foam meniscus

**DOI:** 10.1098/rspa.2022.0133

**Published:** 2022-06

**Authors:** P. Grassia

**Affiliations:** Department of Chemical and Process Engineering, University of Strathclyde, James Weir Building, 75 Montrose Street, Glasgow G1 1XJ, UK

**Keywords:** foam rheology, foam films, surfactant transport, Gibbs elasticity, meniscus, mathematical modelling

## Abstract

A model developed by Bussonnière & Cantat [1] is considered for film-to-film surfactant transport around a meniscus within a foam, with the transport rate dependent upon film-to-film tension difference. The model is applied to the case of a five-film device, in which motors are used to compress two peripheral films on one side of a central film and to stretch another two peripheral films on the central film’s other side. Moreover, it is considered that large amounts of compression or stretch are imposed on peripheral films, and also that compression or stretch might be imposed at high velocities (relative to a characteristic velocity associated with physico-chemical properties of the foam films themselves). The actual strain that results on elements within each film might differ from the imposed strain, with the instantaneous film length coupled to the actual strain determining the amount of surfactant currently on each film (and hence also the amount of surfactant that has transferred either from or onto films). Quite distinct surfactant transport behaviour is predicted for the stretched film compared with the compressed one. In particular, when a film is stretched sufficiently at high enough velocity, surfactant flux onto it is predicted to become extremely ‘plastic’, increasing significantly.

## 1. Introduction

As well as being familiar in everyday life [2–5] and useful in myriad industrial processes [6–12], foams have long held a fascination for physicists. The reason for this fascination is well documented [13,14]: foam films reduce their surface energy by reducing their surface area, meaning that films are minimal surfaces subject to the constraint that bubbles fill certain volumes [15–18].

Similar notions (i.e. constrained minimization of area) can also be used to study foam rheology [19]. Starting from a relaxed configuration, the boundaries of a foam can be subject to deformation, and the foam needs to find new minimal surface states subject to those additional constraints on the boundary [20–24]. Generally, this involves higher energy cost than before. However, for sufficient deformation, it may be the case that certain films shrink away to zero size, so bubbles formerly in contact will detach from one another and other bubbles will come into contact in their place, forming new films [22]. This process, known as a topological transformation [13,25,26], helps the surface energy to relax thereby allowing the foam to yield plastically [20,23,24] and subsequently to approach a new mechanical equilibrium.

Despite the very valuable insights into foam physics and/or foam rheology that can thereby be gained, the body of work described above neglects one important aspect of foams, namely physical chemistry. In fact, aqueous foams are typically produced with the aid of chemical additives, i.e. surfactants [27]. As soon as one starts deforming the foam films, surfactant starts moving around also [28–30]: physico-chemical effects associated with surfactant transport thereby couple with foam flow and foam-film deformation [31–38].

The behaviour of the foam then relies on the interplay between many different time scales [31,32,39–45] involving at the very least a characteristic time scale for imposed deformation, a characteristic time scale for mechanical relaxation of the foam, and a characteristic time scale for physico-chemical relaxation of the foam, i.e. a time scale associated with surfactant transport. If there happen to be multiple mechanisms for surfactant transport [46], there may of course be more than one physico-chemical time scale.

In many ways, these physico-chemical aspects of foams are more difficult to deal with than the physical (i.e. minimal surface) aspects: foam-film geometry can be easily observed directly, but individual surfactant molecules cannot. Hence, information about surfactant behaviour must be inferred from other measurements [47,48], often using techniques that determine surface tension [49,50]. What is known is that stretching the film depletes the amount of surfactant on the surface, leading to an increase in film tension [51], which is a phenomenon known as Gibbs elasticity [52].

Although the influence of surfactants upon foam-film surfaces is well documented, these physical chemistry studies have very often focussed upon geometrically simple systems: either the interface of a single droplet or a single bubble or indeed just a single film [28,47,53–55]. While studies like these give valuable insights into surfactant transport within and along individual film surfaces, they still fall short of understanding how surfactant is transported within a foam as a whole. Indeed, one mechanism that studies on individual films cannot capture, but which must be relevant in foams (albeit neglected in at least one previous study [38] owing to lack of an adequate model), is surfactant transport from a foam film to its neighbouring films [56].

This mechanism certainly will be relevant in the aforementioned topological transformation process [13,20,23]. Films that shrink and then disappear on the approach to the topological transformation presumably must transport surfactant over to their neighbours. Meanwhile, new films that are created during the topological transformation start out with a small size, but are necessarily stretched as the foam then relaxes mechanically. Stretching in isolation would cause the film to deplete in surfactant, but this can be compensated if it receives surfactant from its neighbours. Specifically, this requires a surfactant flux around the menisci joining any neighbouring films to the newly formed film of interest.

There have been various hypotheses [57–59] for what the surfactant flux should be around such menisci, all based on the idea that Marangoni effects drag material from low tension (high surfactant concentration on the surface) to high tension (low surfactant concentration on the surface). Often, though, hypotheses like these have been largely empirical. Nevertheless, a recent study [1] (see also [60] for a brief commentary thereon) including both a rigorous fluid mechanical analysis and an experimental study (using the same apparatus already introduced in [56]) has now placed these sorts of hypotheses on a firmer basis.

The fluid mechanical analysis of [1] identified clearly the main challenge with determining film-to-film surfactant fluxes, namely so-called geometrical frustration. At a meniscus, also known in the context of foams as a Plateau border [13], three films meet. As a result, a film that is passing surfactant to or receiving surfactant from one of its neighbours only communicates with that neighbour on one side. The other side of the film in question contacts a different neighbour, possibly in a very different state from the neighbour on the original side, and hence possibly involving a different amount of surfactant transport. Close to a meniscus, a model therefore ought somehow to account for different sides of an individual film having different amounts of surfactant (a point we will return to in §2biii), even though further away from the meniscus, such differences are less significant.

**Figure 1.**
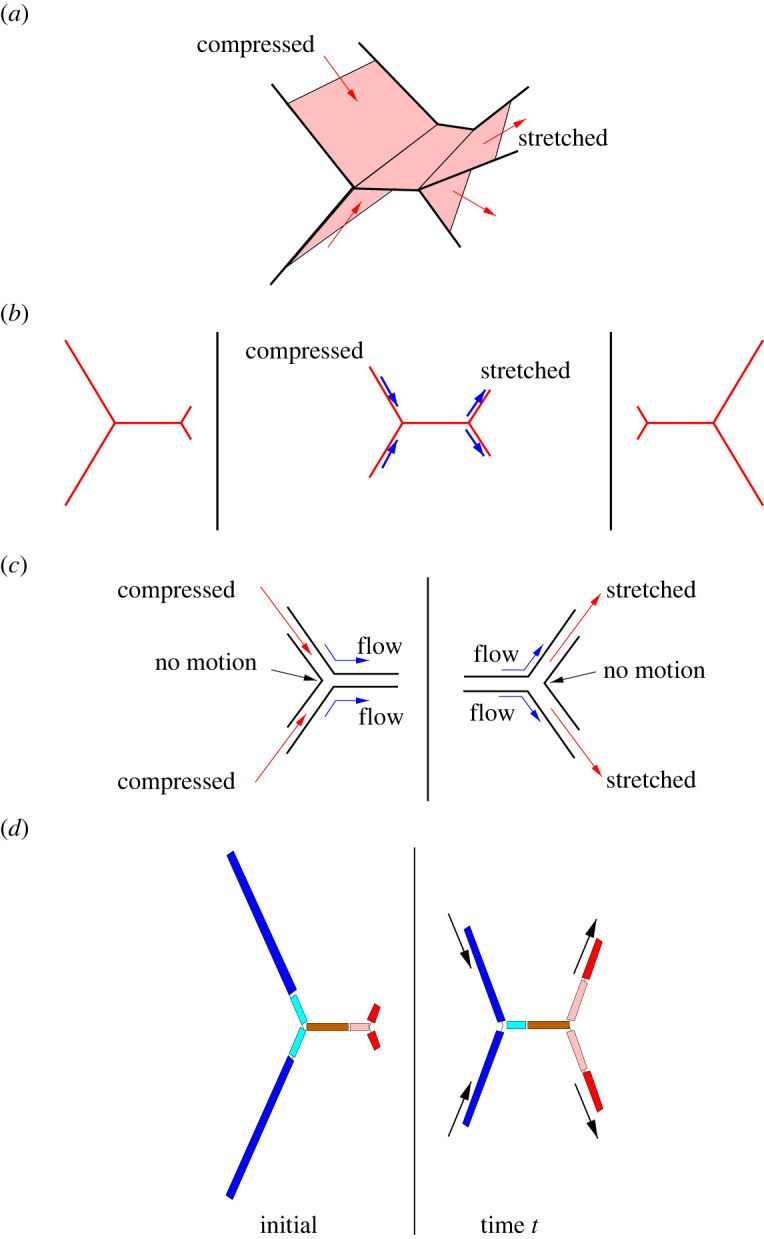
Sketch of the five-film device. (*a*) Three-dimensional view. (*b*) Two-dimensional view showing initial state (left), stretching or compression by a motor (middle), and the state after motor motion is complete (right). (*c*) Zoomed view close to the meniscus for the compressed film (left) and stretched film (right). This shows geometrical frustration, i.e. close to the meniscus, it is not possible to have the same flow on both sides of every film. (*d*) Lagrangian film elements (colour-coded), which are used to determine the amount of surfactant on each film in the five-film device. It is first identified (via the colour-coding) which Lagrangian elements are on a given film at a given time t subject to compression or stretch (right), and then the length that those elements had initially, prior to any compression or stretching, is determined (left), from which the amount of surfactant is then known. (Online version in colour.)

Meanwhile, the experimental system studied by Bussonnière & Cantat [1], while not quite the same as what happens during a topological transformation, nor anywhere near as complex as a general foam, is nevertheless far more complex than just a single film would be. Specifically, it was a five-film device (figure 1) with a central film connecting to four peripheral films, two on each side of the central film. The system could be driven by motors, such that the two peripheral films on one side of the central film are compressed, and the two peripheral films on the other side are stretched. Surfactant could then be transported from the compressed film to the central film and from the central film to the stretched film (more detail on this is given in §2a). However, such transport is affected, as already alluded to above, by geometrical frustration (figure 1*c*).

The system permitted a constitutive relation between film-to-film tension difference and surfactant flux around the meniscus [1] to be proposed and tested (detailed discussion of the relation itself is deferred to §2b). Using this constitutive relation, equations were proposed (again detailed discussion is given later, namely in §2c) governing the evolution of how much surfactant finds its way from film to film, both while motors are switched on and after they are switched off. However, these equations were only solved by Bussonnière & Cantat [1] in a limited scenario in which just small amounts of compression or stretch were imposed (i.e. in a limit of small imposed strains), and hence just small amounts of surfactant were transferred. Solutions for the surfactant transport involving simple exponential decays then resulted.

The constitutive relation developed by Bussonnière & Cantat [1] was not itself restricted to small imposed strains. It is merely the case that when [1] developing a model for the five-film device as a whole (the constitutive relation being just one of the ingredients of that model), solutions were only tackled in the limit of small imposed compression or stretch. The purpose of the present work is to return to the model of [1] but then impose larger amounts of compression or stretch, beyond the regime of validity of the aforementioned simple exponential solutions. As we will see, surfactant manages to escape from the compressed film, without accumulating excessively, even if the compression is very strong. Meanwhile for the stretched film, if a significant amount of stretch is imposed at a sufficient rate, the constitutive relation allows surfactant to be transported rather more quickly onto the film. Even though the present work treats only the five-film device of [1] and not a general foam, gaining insights into behaviours of films subject to large amounts of compression or stretch is deemed of interest: the aforementioned topological transformations occurring in a general foam do of course involve films subject to large amounts of compression or stretch.

The rest of this work is laid out as follows. In §2, we review the constitutive relation of [1] and surfactant transport equations that it implies. In §3, we explain how to solve these equations even in a scenario of large imposed strains. Results obtained from these solutions are presented/discussed in §4. Conclusions are given in §5. Some technical details of both the model and the solution procedure are relegated to appendices (in electronic supplementary material).

## 2. Model and governing equations

In this section, we review the model and governing equations proposed by Bussonnière & Cantat [1]. The presentation is given within three subsections. The first of these (§2a) looks at surfactant transport around a meniscus. Then §2b considers how to relate that transport to film-to-film tension differences. After that, §2c then shows how to incorporate strains that are imposed on various films by the action of motors. In particular, §2c is formulated in a fashion that lends itself to dealing with large strains, involving a slightly different way of expressing the model than the formulation that [1] used. The formulations are nonetheless equivalent. Readers already familiar the work of [1] may wish to skip directly to §2c and in particular equation (2.13), which expresses the model in a compact and elegant form.

### (a) Surfactant transport around a foam meniscus

Considering the compressed film in the first instance, the amount of surfactant on the film at any instant is represented by a quantity $L_{0}^{-}$, which is defined as follows. Accounting just for the film elements that are currently on the compressed film, not those that have already been transferred from the compressed film to the central film (see figure 1*d* for an illustration), the value of $L_{0}^{-}$ represents the length that these elements had before the structure was set into motion. If we happen to know the instantaneous velocity $U^{-}$ at the meniscus (in the direction from the compressed film to the central film), then we can determine how $L_{0}^{-}$ evolves with time t. Specifically, it was proposed [1]

2.1 $$\frac{\text{d}L_{0}^{-}}{\text{d}t}=\frac{-U^{-}}{\left(1+\varepsilon^{-}\right)},$$

where $\varepsilon^{-}$ is the instantaneous strain in the compressed film upstream of the meniscus. Here, $\varepsilon^{-}$ is negative ($-1<\varepsilon^{-}<0$), i.e. the film is compressed. Thus, $|\text{d}L_{0}^{-}/\text{d}t|$ exceeds the value of $U^{-}$, which follows because, for a given velocity at the meniscus, a greater surfactant flux results if surfactant has been concentrated due to the film elements having been already compressed.

An analogous relationship applies for the stretched film

2.2 $$\frac{\text{d}L_{0}^{+}}{\text{d}t}=\frac{U^{+}}{\left(1+\varepsilon^{+}\right)},$$

which we interpret as follows. The value of $L_{0}^{+}$ refers to the original length (before the structure was set into motion) of all the film elements that are currently on the stretched film. Not all these elements will have originated on the stretched film, however. Instead, some of them might have arrived from the central film (again, see figure 1*d* for an illustration). Moreover, the instantaneous velocity $U^{+}$ is defined at the meniscus in the sense from the central film to the stretched film. Also, the strain $\varepsilon^{+}$ in the stretched film downstream of the meniscus is positive. A consequence is that $\text{d}L_{0}^{+}/\text{d}t$ is smaller than $U^{+}$. For a given velocity at the meniscus, surfactant flux is less if surfactant is depleted due to having stretched film elements.

To complete the model of [1], it is necessary to provide constitutive relations to determine the velocities $U^{-}$ and $U^{+}$. These are discussed in the next section.

### (b) Constitutive relations for velocities at the menisci

Velocities $U^{-}$ and $U^{+}$ at the menisci were taken by Bussonnière & Cantat [1] to be functions of the differences in tensions between adjacent films (with film tensions being twice the surface tension here, as films have two sides). Accordingly, differences in film tensions are discussed in §2bi. The amounts of surfactant flux that these tension differences then manage to produce are discussed in §2bii (compressed case) and §2biii (stretched case). As we will see, the stretched case turns out to be somewhat more complicated than the compressed one.

#### (i) Differences in film tensions

The tension differences required are between the central film and the compressed film, and between the stretched film and the central film, denoted as $\Delta_{-c}\sigma$ and $\Delta_{c+}\sigma$, respectively. Both of these tension differences (central to compressed, and stretched to central) are expected to be positive because the compressed film should have the lowest tension of all, the central film should have an intermediate tension, and the stretched film should have the highest tension. It was argued by Bussonnière & Cantat [1] that on symmetry grounds the central film does not undergo either stretching or compression to any significant degree, and hence is assumed to have a tension that is always close to an equilibrium tension $\sigma_{e}$. It follows then that

2.3 $$\Delta_{-c}\sigma =-\Delta_{e}\sigma^{-}$$

and

2.4 $$\Delta_{c+}\sigma =\Delta_{e}\sigma^{+},$$

where $\Delta_{e}\sigma^{\pm }$ is the deviation from equilibrium tension in either the compressed or stretched film. This should be negative in the compressed film but positive in the stretched film, i.e. it should have the same sign as the film strain $\varepsilon^{\pm }$. A Gibbs elasticity model was then invoked by Bussonnière & Cantat [1] relating the film tension deviation from equilibrium to the film strain. This gave

2.5 $$\Delta_{e}\sigma^{\pm }=\frac{2E\;\varepsilon^{\pm }}{\left(1+\varepsilon^{\pm }\right)},$$

where the factor 2 recalls that films have two sides, and where E is the Gibbs elasticity parameter. Although the value of E could in principle itself vary with strain, it was treated by Bussonnière & Cantat [1] as being constant. As we will see (in §2c), this turns out to be convenient mathematically as it makes the system somewhat easier to solve. Physically, what equation (2.5) means is as follows: although $\Delta_{e}\sigma^{\pm }$ is clearly nonlinear in $\varepsilon^{\pm }$, if it is plotted instead against the surfactant concentration on the films, a straight line plot then results [1] as surfactant concentration turns out to scale inversely with $1+\varepsilon^{\pm }$, and also $\Delta_{e}\sigma^{\pm }\equiv 2E-2E/(1+\varepsilon^{\pm })$.

Moreover in the experimental work carried out by Bussonnière & Cantat [1], film strains could be determined by measuring film thickness (using interferometric techniques): the more film elements are stretched, the thinner they become. Meanwhile, film tension differences could be determined experimentally by measuring the angles at which films meet at a meniscus, with these angles being found in turn via the displacement of the meniscus relative to its equilibrium position: films meeting at unequal angles imply unequal tension. Since film strains and film tensions can thereby be determined via the above-mentioned measurements, and the values thus obtained compared with equation (2.5), experimental support is available for using equation (2.5) with E treated as constant. As alluded to earlier, this makes the system easier to solve.

To summarize, we reiterate the signs of various terms. Recalling that $\varepsilon^{-}<0$ in the compressed film, it follows from equation (2.5) that $\Delta_{e}\sigma^{-}<0$, i.e. tension is less than equilibrium as we expect. On the other hand, for the stretched film $\varepsilon^{+}>0$, so that $\Delta_{e}\sigma^{+}>0$, i.e. tension exceeds equilibrium. Via equations (2.3)–(2.4), it then follows that $\Delta_{-c}\sigma$ and $\Delta_{c+}\sigma$ have the correct signs (both positive) to drive surfactant flux around the menisci in the expected directions (from compressed film to central film and from central film to stretched film), and so we can proceed to consider constitutive relations expressed in terms of these tension differences.

#### (ii) Constitutive relation for compressed film

In the first instance, the compressed film is considered. By examining numerous sets of data, it was found [1] that there was a proportionality relationship between $U^{-}$ and $\Delta_{-c}\sigma$, or equivalently a proportionality relationship between $U^{-}$ and a dimensionless quantity $\Delta_{-c}\sigma /(2E)$, remembering here that E is treated as being constant. The coefficient of proportionality, which has units of velocity, was denoted $U^{*}$. This coefficient can be thought of as a property of the foam film, but is independent of how quickly the film is compressed. A formula was also proposed [1] for how $U^{*}$ should depend on various physico-chemical properties of the film (see electronic supplementary material, appendix A), and by using it, different datasets could be collapsed together well. In the case of the compressed film, proportionality between $U^{-}$ and $\Delta_{-c}\sigma /(2E)$ could continue to apply, even for $\Delta_{-c}\sigma /(2E)$ values well in excess of unity, i.e. $U^{-}$ well in excess of $U^{*}$. The only restriction was an obvious physical one, i.e. the central film remains by assumption at equilibrium tension while the compressed film can have a much smaller tension, so that $\Delta_{-c}\sigma$ can never exceed the equilibrium tension (denoted $\sigma_{e}$) but may well exceed 2E.

Given that tension difference and strain are related via equation (2.5), it turns out to be convenient to rewrite $U^{-}$ in terms of strain, remembering also here that $\varepsilon^{-}$ is a negative quantity. Via equations (2.3) and (2.5), it follows:

2.6 $$U^{-}=\frac{-U^{*}\varepsilon^{-}}{\left(1+\varepsilon^{-}\right)},$$

and thence (via equation (2.1))

2.7 $$\frac{\text{d}L_{0}^{-}}{\text{d}t}=\frac{U^{*}\varepsilon^{-}}{{\left(1+\varepsilon^{-}\right)}^{2}}.$$

We will revisit this equation later on (see equation (2.12)). Note that [1] did not formally derive this equation in the specific form shown here, but it turns out to be very useful in the large strain limit that we will consider later on.

#### (iii) Constitutive relation for stretched film

Now we return to the stretched film. Examining numerous sets of data, a proportionality relationship was again found [1] between $U^{+}$ and $\Delta_{c+}\sigma /(2E)$, with the proportionality coefficient being the same $U^{*}$ as before. This led via analogous arguments using equation (2.4) and equation (2.5) to

2.8 $$U^{+}=\frac{U^{*}\varepsilon^{+}}{\left(1+\varepsilon^{+}\right)},$$

and thence (via equation (2.2))

2.9 $$\frac{\text{d}L_{0}^{+}}{\text{d}t}=\frac{U^{*}\varepsilon^{+}}{{\left(1+\varepsilon^{+}\right)}^{2}},$$

which, although again being an equation not formally derived by Bussonnière & Cantat [1], will be useful later on.

It was argued by Bussonnière & Cantat [1] that the relationship between meniscus velocity and tension difference in the stretched case is more constrained than before. The value of $\Delta_{c+}\sigma /(2E)$ (which via equation (2.4) is equal to $\Delta_{e}\sigma^{+}/(2E)$) was not allowed to exceed 1/2, or equivalently (via equation (2.5)), $\varepsilon^{+}$ was not allowed to exceed unity. If $\varepsilon^{+}$ ever did attain the value unity, then $U^{+}$ would take whatever value was needed, even a value well in excess of the prediction $U^{*}/2$ now obtained from equation (2.8), in order to prevent $\varepsilon^{+}$ from exceeding unity and thereby in turn preventing $\Delta_{e}\sigma^{+}/(2E)$) from exceeding 1/2. Thus, the strain $\varepsilon^{+}=1$ corresponds to the flow $U^{+}$ at the meniscus suddenly becoming very plastic, which then helps to transport surfactant onto the stretched film.

At first sight, it seems surprising that the system becomes plastic when $\Delta_{e}\sigma^{+}/(2E)$ reaches the value 1/2. After all, according to equation (2.5), the value $\Delta_{e}\sigma^{+}/(2E)$ could reach up to unity for an even more strongly stretched film ($\varepsilon^{+}\gg 1$). However, the explanation why $\Delta_{e}\sigma^{+}/(2E)$ is capped at 1/2 has been provided by Bussonnière & Cantat [1] as being due to geometrical frustration (already alluded to in the introduction). Conventionally, we think of the two sides of a film as being the same in terms of their surface tension, so the film tension is exactly twice the surface tension on either side, and this is indeed true over most of the film length. Nevertheless, sufficiently close to the meniscus this rule does not apply (figure 1*c*). Locally, flow might be different on different sides of a film and that impacts surface tension. Film tension remains the sum of the surface tensions either side of the film, albeit the tensions either side need not be the same.

For instance, in the five-film device (see figure 1 and in particular figure 1*c*), one side of the stretched film communicates with the central film (and can receive a supply of surfactant from it), while the other side is in contact only with another stretched film. This latter side is then the one that can deplete in surfactant but, as a result of even very strong depletion, at most its individual surface tension can increase by an amount E relative to equilibrium, at least in the model of [1] assuming a constant Gibbs elasticity parameter. This then also sets the cap on the amount that film tension can increase above equilibrium. Moving slightly away from the meniscus, it was explained by [1] that the two surfaces of the film adjust (while keeping the same overall film tension) such that the surface tension on each side (and likewise the surface strain on each side) come into balance, and that then is the strain used to determine the film tension in equation (2.5). The important point as far as the present work is concerned is then as follows. As long as the correct cap on film tension and hence on film strain is applied, the constitutive equation determining the film-to-film surfactant flux can be used, even without having full details of the geometrical frustration-induced behaviour at the meniscus itself.

### (c) Model incorporating imposed strains in five-film device

Using the constitutive relations described above that govern $U^{-}$ and $U^{+}$, a model could be proposed [1] for the behaviour of the five-film device. The model developed (see equation (2.13) below) is a ‘lumped parameter’ model. What this means is that it treats the strain in the compressed or stretched films as being spatially uniform (at least away from the meniscus) but different from the strain in the central film, which (as already alluded to in §2b) by assumption vanishes. We comment that in what follows, the equations are presented slightly differently from the way in which [1] chose to present them: specifically, we work in terms of film strains rather than film tensions. This slightly different (albeit mathematically equivalent) presentation helps to elucidate the mathematical structure of the model, which then makes it clearer how to solve it (see §3), particularly when large strains are involved as we consider here. As mentioned already, even though the constitutive relation of [1] can cope with large strains, the lumped parameter model for the five-film device itself was only tackled by Bussonnière & Cantat [1] in the small strain limit.

The model begins from the definition of the ‘lumped’ strain on the films

2.10 $$L^{\pm }=(1+\varepsilon^{\pm })L_{0}^{\pm },$$

where $L^{\pm }$ is the instantaneous length of either the compressed or stretched film, and $\varepsilon^{\pm }$ and $L_{0}^{\pm }$ are as given previously. We differentiate this with respect to time t to obtain

2.11 $$\frac{\text{d}L^{\pm }}{\text{d}t}=(1+\varepsilon^{\pm })\frac{\text{d}L_{0}^{\pm }}{\text{d}t}+L_{0}^{\pm }\frac{\text{d}\varepsilon^{\pm }}{\text{d}t}.$$

We now eliminate terms in $L_{0}^{\pm }$ and $\text{d}L_{0}^{\pm }/\text{d}t$ from the right-hand side of equation (2.11). Using equation (2.10), it follows $L_{0}^{\pm }=L^{\pm }/(1+\varepsilon^{\pm })$. Meanwhile, equations (2.7) and (2.9) give us the expression for $\text{d}L_{0}^{\pm }/\text{d}t$, which is

2.12 $$\frac{\text{d}L_{0}^{\pm }}{\text{d}t}=\frac{\pm U^{\pm }}{\left(1+\varepsilon^{\pm }\right)}=\frac{U^{*}\varepsilon^{\pm }}{{\left(1+\varepsilon^{\pm }\right)}^{2}},$$

noting however that in the case of stretching in particular, the second equality in equation (2.12) is only valid when $\varepsilon^{+}<1$. If instead $\varepsilon^{+}$ reaches the value unity and stays fixed there, equation (2.10) gives $\text{d}L_{0}^{+}/\text{d}t=(1/2)\text{d}L^{+}/\text{d}t$. In the case when $\varepsilon^{+}=1$, equation (2.2) gives $\text{d}L_{0}^{+}/\text{d}t=U^{+}/2$ and hence it follows $U^{+}=\text{d}L^{+}/\text{d}t$. This relation holds instead of $U^{+}$ satisfying equation (2.8), which for $\varepsilon^{+}=1$ would give instead a value of $U^{*}/2$. Thus, as long as $\text{d}L^{+}/\text{d}t$ exceeds $U^{*}/2$, a jump in $U^{+}$ must occur once $\varepsilon^{+}=1$. The surfactant transport rate $\text{d}L_{0}^{+}/\text{d}t$ likewise jumps from $U^{*}/4$ to the aforementioned value $(1/2)\text{d}L^{+}/\text{d}t$ once $\varepsilon^{+}=1$.

In the first instance though, we consider $\varepsilon^{+}<1$. Upon multiplying equation (2.11) through by $1+\varepsilon^{\pm }$, and substituting from equation (2.12), this leads to

2.13 $$(1+\varepsilon^{\pm })\frac{\text{d}L^{\pm }}{\text{d}t}=U^{*}\varepsilon^{\pm }+L^{\pm }\frac{\text{d}\varepsilon^{\pm }}{\text{d}t}.$$

Now, in the five-film device, $L^{\pm }$ is a known function of t. Specifically, if $L_{i}^{\pm }$ is the initial film length (which generally is not the same for the compressed and stretched films, see e.g. figure 1*b*), and V is the velocity at which the motor compresses or stretches the films, we have

2.14 $$L^{\pm }=L_{i}^{\pm }\pm V\;t$$

from which it also follows $\text{d}L^{\pm }/\text{d}t=\pm V$. Note that the strain imposed on the films by the motor is $(L^{\pm }-L_{i}^{\pm })/L_{i}^{\pm }\equiv \pm V\;t/L_{i}^{\pm }$. However, owing to film-to-film surfactant transport, this imposed strain is in general different from the strain $\varepsilon^{\pm }$ that develops on the films themselves, as given by equation (2.10).

An important observation is that equation (2.13) now takes the form of an inhomogeneous, linear, first-order differential equation, albeit with variable coefficients, and this is the basis upon which we can solve it. The fact that the equation still turns out to be linear, despite large strains being imposed on the films, relies in turn upon the film tension model in equation (2.5), and also the linear relation between meniscus velocity and deviation in film tension. Solutions of the system as obtained by Bussonnière & Cantat [1] assumed small imposed strains or equivalently $V\;t\ll L_{i}^{\pm }$, so in effect approximated the variable coefficient model by a constant coefficient one, leading to solutions in terms of exponentials. However, that approximation will not be employed here.

Even without that approximation, there is still a scenario in which a constant coefficient case is recovered. The motor is only actually switched up to some time $t_{m}$. After that, $L^{\pm }$ is held fixed at a value $L_{m}^{\pm }\equiv L_{i}^{\pm }\pm V\;t_{m}$ whereas $\text{d}L^{\pm }/\text{d}t$ vanishes: equation (2.13) then reverts to having constant coefficients. Thus, the model involves an initial motor-driving phase followed by a subsequent relaxation phase, and solutions for both of these phases are required. The way in which to obtain solutions is outlined next.

## 3. Obtaining solutions of the model

Details of how to solve the model are given in electronic supplementary material, appendix B, so only a brief outline will be given here. Solutions of the model are more conveniently expressed in dimensionless form (see electronic supplementary material, appendix B(a)). Specifically, we define dimensionless film lengths $L^{\pm }=L^{\pm }/L_{i}^{\pm }$ and also dimensionless measures of the amount of surfactant on the films $L_{0}^{\pm }=L_{0}^{\pm }/L_{i}^{\pm }$, along with dimensionless times $\tau^{\pm }=U^{*}t/L_{i}^{\pm }$. The dimensionless compression or stretch velocity v is defined as $v=V/U^{*}$. As electronic supplementary material, appendix A, explains, typical values of $U^{*}$ (a physico-chemical parameter of the foam films) are well within the range of velocities V that a typical motor could attain. Hence, it is possible to contemplate v≪1 for a motor operating far below its maximum velocity, but also v≫1 for a motor operating closer to maximum velocity. Strains $\varepsilon^{\pm }$ are of course already dimensionless. However, it is important to remember that $\varepsilon^{\pm }$ here denotes the actual strain developed on the film elements themselves, accounting for film-to-film surfactant transport. As already mentioned (see §2c), these strains differ from the strains imposed on the films by the action of the motors, which turn out to be $\pm V\;t/L_{i}^{\pm }$ or equivalently (in dimensionless variables) $\pm v\tau^{\pm }$. Indeed, the actual strains only become the same as the imposed strains when film-to-film surfactant transport is neglected. In general, however, during motor motion, actual strains turn out to be smaller in magnitude than the imposed ones.

From here onward, we work in terms of dimensionless variables. With the above definitions for these variables, solutions of the model can now be obtained (see electronic supplementary material, appendix B(b)). The method for obtaining solutions relies on replacing the dimensionless time $\tau^{\pm }$ by a modified time $T^{\pm },\langle T^{\pm }\rangle ,$ which depends on both $\tau^{\pm }$ and v: within electronic supplementary material, appendix B(b), see equations (B.13) and (B.18) along with figures B1 and B2. These equations for $T^{\pm }$ are reproduced below

3.1 $$T^{-}=-\frac{1}{|\varepsilon_{l}^{-}|}\log(1-v\;\tau^{-})$$

and

3.2 $$T^{+}=\frac{1}{\varepsilon_{l}^{+}}\log(1+v\tau^{+}).$$

These equations involve a limiting strain $\varepsilon_{l}^{\pm }$, which is the actual strain on the films that would be realized in the limit of large $T^{\pm }$, corresponding to significant amounts of either compression or stretch being imposed, albeit in the stretching case not yet accounting for flux possibly becoming plastic. The values of $\varepsilon_{l}^{\pm }$ (see electronic supplementary material, equations (B.11) and (B.16)) depend on the velocity v, with smaller v leading to smaller magnitude limiting strain. These equations for $\varepsilon_{l}^{\pm }$ are reproduced below

3.3 $$\varepsilon_{l}^{-}=\frac{-v}{\left(1+v\right)}$$

and

3.4 $$\varepsilon_{l}^{+}=\frac{v}{\left(1-v\right)}.$$

In the compressed case, the value of $\varepsilon_{l}^{-}$ turns out to be negative, so we often write $-|\varepsilon_{l}^{-}|$ to make its sign explicit. Meanwhile, in the stretched case, the formula for $\varepsilon_{l}^{+}$ becomes problematic as v approaches and eventually exceeds unity. Solutions of the model then need to take a slightly different form in that case: details are given in electronic supplementary material, appendix C.

When written in terms of modified time $T^{\pm }$, it turns out that the variable coefficient differential equation (2.13) converts to a constant coefficient one, so even though imposed strains no longer need to be small, solutions just involve exponentials but in terms of $T^{\pm }$ not $\tau^{\pm }$: see electronic supplementary material, equations (B.14) and (B.19). These equations for $\varepsilon^{\pm }$ are reproduced below

3.5 $$\varepsilon^{-}=-|\varepsilon_{l}^{-}|(1-\exp(-T^{-}))$$

and

3.6 $$\varepsilon^{+}=\varepsilon_{l}^{+}(1-\exp(-T^{+})).$$

As has been mentioned already, in the compressed case, strains are negative. In the stretched case, strains are positive, but we can encounter circumstances in which $\varepsilon^{+}$ reaches the value of unity, at which point film-to-film surfactant transport becomes very plastic (as §2biii explained). This occurs at a modified time that we denote $T_{\mathrm{pl}}^{+}$ or equivalently at an imposed strain that we denote $v\tau_{\mathrm{pl}}^{+}$. Formulae for $T_{\mathrm{pl}}^{+}$ and $v\tau_{\mathrm{pl}}^{+}$ are easy to derive starting from equations (3.6) and (3.2) (equivalently, electronic supplementary material, equations (B.19) and (B.18)). The relevant formulae are given in electronic supplementary material, equations (B.21) and (B.22), and ultimately just depend on v. As one might expect, increasing v causes $\varepsilon^{+}$ to attain the value of unity sooner, as will be verified shortly.

The above-mentioned approach defining a modified time $T^{\pm }$ is only needed as long as motors are driving the system. Motors are, however, stopped at some well-defined time $\tau_{m}^{\pm }$ or equivalently some well-defined imposed strain $v\tau_{m}^{\pm }$, corresponding to film lengths $L_{m}^{\pm }$ and instantaneous strains in the films $\varepsilon_{m}^{\pm }$. The system is then allowed to relax by exchanging surfactant even though film lengths are now held fixed. A solution for $\varepsilon^{\pm }$ directly in terms of $\tau^{\pm }$ is then available. Again, exponential solutions occur: see electronic supplementary material, equation (B.23) within electronic supplementary material, appendix B(b)iii. This equation is also reproduced below

3.7 $$\varepsilon^{\pm }=\varepsilon_{m}^{\pm }\exp\left(-\frac{\left(\tau^{\pm }-\tau_{m}^{\pm }\right)}{L_{m}^{\pm }}\right).$$

Once $\varepsilon^{\pm }$ is known via either equations (3.5), (3.6), or (3.7), it is simple to find the amount of surfactant on films $L_{0}^{\pm }$, using the value of $\varepsilon^{\pm }$ and also the film length $L^{\pm }$. The equations needed are dimensionless analogues of equations (2.10) and (2.14): see electronic supplementary material, equations (B.2)–(B.3) and (B.5)–(B.6).

This completes our brief outline of how solutions are obtained. Results showing how the various solutions behave are described next.

## 4. Results

This results section is divided into three subsections. The first of these presents results for the compressed film case (§4a), while the second presents the stretched film case (§4b). Finally, systems are considered that are allowed to relax after being either compressed or stretched (§4c).

### (a) Compressed film

In the compressed film case, we present data first for the strain on film (§4ai) and then for the resulting amount of surfactant on the film (§4aii).

#### (i) Strain during compression

**Figure 2.**
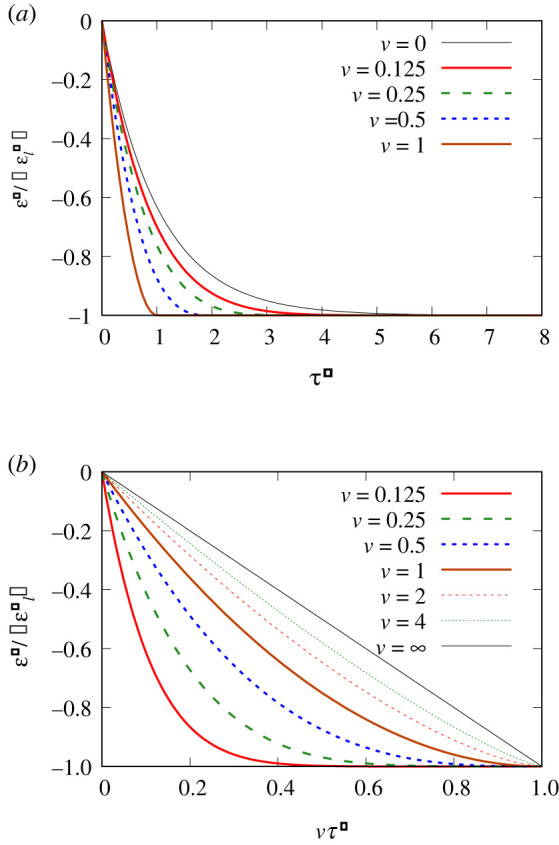
Compressed film: (*a*) $\varepsilon^{-}/|\varepsilon_{l}^{-}|$ versus $\tau^{-}$, and (*b*) $\varepsilon^{-}/|\varepsilon_{l}^{-}|$ versus $v\tau^{-}$. (Online version in colour.)

Data for compressive strains $\varepsilon^{-}$ (or more precisely, for $\varepsilon^{-}$ relative to $|\varepsilon_{l}^{-}|$) are presented in figure 2. Specifically, in figure 2*a*, we plot this against dimensionless time $\tau^{-}$, although an indication of what this represents in terms of dimensional time is discussed in electronic supplementary material, appendix D. Note that (see electronic supplementary material, figure B.1*a* in appendix B) for a given time $\tau^{-}$, increasing v leads to an increased $T^{-}$, and hence a $\varepsilon^{-}$ that (according to equation (3.5) or equivalently electronic supplementary material, equation (B.14)) in relative terms is closer to the limiting strain $-|\varepsilon_{l}^{-}|$. This then is what we see in figure 2*a*. The case that is in relative terms furthest away from $-|\varepsilon_{l}^{-}|$ for longest is the limiting case v→0, corresponding to a very slow stretch. In this particular case, $\varepsilon^{-}$ takes quite some time to evolve from zero to $-|\varepsilon_{l}^{-}|$. However, in fact, $\varepsilon^{-}$ barely changes at all in absolute terms, because $|\varepsilon_{l}^{-}|$ is itself vanishingly small in the v→0 limit.

Rather than plotting data against $\tau^{-}$, it is also possible to plot against $v\tau^{-}$ (figure 2*b*). As has been mentioned, physically $v\tau^{-}$ represents an imposed strain, i.e. the length change imposed on the compressed film divided by its initial length. For a given $v\tau^{-}$ (i.e. a given imposed strain), increasing v leads to a smaller $T^{-}$ (see electronic supplementary material, figure B.1*b* in appendix B) and hence an $\varepsilon^{-}$ that in relative terms is further away from $-|\varepsilon_{l}^{-}|$. That is what we see in figure 2*b*. Note, however, that electronic supplementary material, figure B.1*b*, also shows that for $v\tau^{-}$ close to unity (correspondingly, according to electronic supplementary material, equation (B.2), to a film compressed to a tiny fraction of its initial length), the value of $T^{-}$ is always very large, regardless of the value of v. In that limit then (again, according to equation (3.5) or equivalently, electronic supplementary material, equation (B.14)), $\varepsilon^{-}$ always approaches $-|\varepsilon_{l}^{-}|$ for any v, which again is seen in figure 2*b*.

#### (ii) Surfactant on film during compression

**Figure 3.**
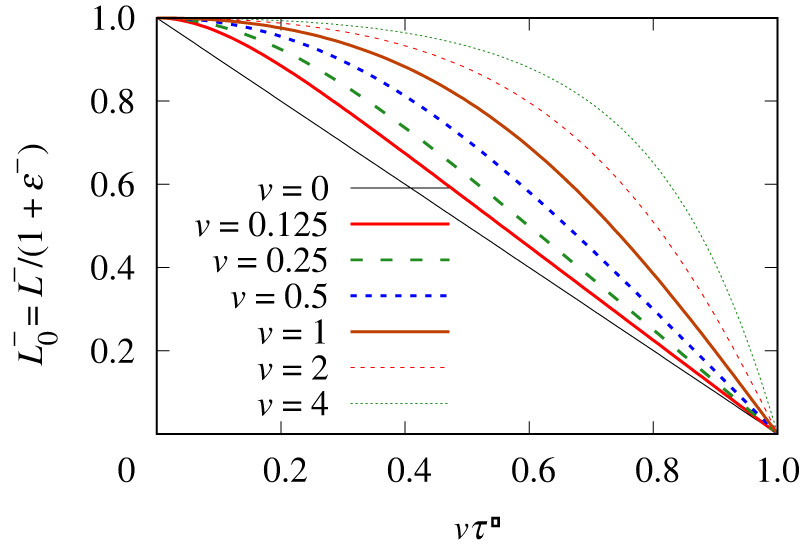
Compressed film: $L_{0}^{-}\equiv L^{-}/(1+\varepsilon^{-})$ versus $v\tau^{-}$. (Online version in colour.)

In figure 3, we show the evolution of $L_{0}^{-}\equiv L^{-}/(1+\varepsilon^{-})$. This measures the amount of surfactant on the compressed film, or more specifically, in the dimensionless system considered here, it measures the amount of surfactant on the compressed film at any given instant relative to the amount that was on the film initially. Clearly, the evolution of $L_{0}^{-}$ is sensitive to the imposed velocity v: to evaluate it, equation (3.5) (equivalently, electronic supplementary material, equation (B.14)) is required, along with electronic supplementary material, equation (B.2). In all cases, the initial rate of change of $L_{0}^{-}$ vanishes (which follows since $\varepsilon^{-}$ vanishes initially, meaning there is no film tension difference to drive surfactant transport). However, in cases with small v, we see $L_{0}^{-}$ start to change significantly even after small imposed strains (i.e. even for relatively small $v\tau^{-}$) and after that $L_{0}^{-}$ tends to follow the evolution of the film length $L^{-}$: indeed, in the case v→0, the value of $L_{0}^{-}$ always equals $L^{-}$ for any specified imposed strain. On the other hand, for larger v, the value of $L_{0}^{-}$ remains high even out to imposed strains $v\tau^{-}$ that are not so far from unity. However, even for these larger v values, in the limit as $v\tau^{-}\to 1$ (such that, according to electronic supplementary material, equation (B.2), the compressed film length $L^{-}$ becomes exceedingly small), we also see small $L_{0}^{-}$ values, i.e. all surfactant can be eventually driven off the film, rather than simply accumulating on it.

### (b) Stretched film

Now we turn to the stretched case, starting off by presenting data for the strain (§4bi). Strains can, however, sometimes become large enough for the surfactant flux to become plastic, so conditions for this to occur are considered in §4bii. The amount of surfactant on the stretched film is analysed in §4biii.

#### (i) Strain during stretching

**Figure 4.**
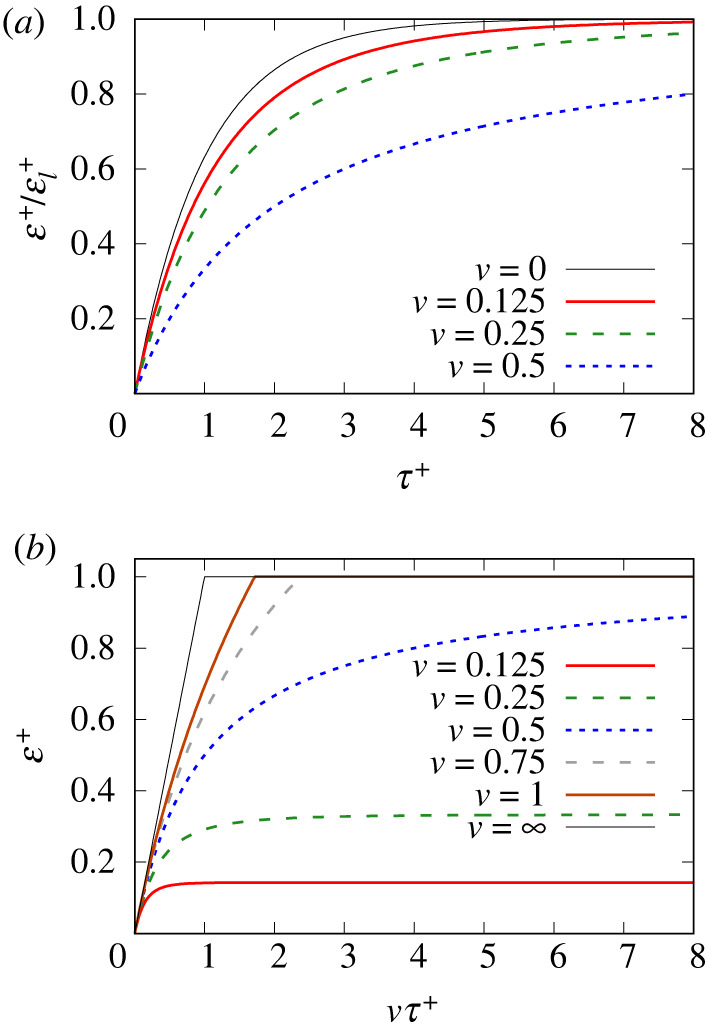
Stretched film: $\varepsilon^{+}/\varepsilon_{l}^{+}$ versus $\tau^{+}$ (*a*) for stretching velocities v≤1/2 only, and (*b*) $\varepsilon^{+}$ versus $v\tau^{+}$ including some larger v values. (Online version in colour.)

In the case of a film subjected to stretching, data for strain $\varepsilon^{+}$ are shown in figure 4. Data in figure 4*a* are plotted against dimensionless time $\tau^{+}$, although electronic supplementary material, appendix D, indicates what this represents in dimensional time. For any fixed $\tau^{+}$, it is found that increasing the value of v decreases the value of $T^{+}$ (see electronic supplementary material, figure B.2 in appendix B) and hence (according to equation (3.6) or equivalently electronic supplementary material, equation (B.19)) moves $\varepsilon^{+}$ in relative terms further from the limiting strain $\varepsilon_{l}^{+}$, as seen in figure 4*a*.

As well as plotting against time $\tau^{+}$, it is also possible (figure 4*b*) to plot data against imposed strain $v\tau^{+}$, which physically is the change in stretched film length divided by its initial length. In fact, at fixed $v\tau^{+}$ (i.e. fixed imposed strain), increasing v also tends to decrease $T^{+}$ (as is evident from equation (3.2) along with equation (3.4), or equivalently electronic supplementary material, equation (B.18) along with electronic supplementary material, equation (B.16)). Hence, based on equation (3.6) or equivalently electronic supplementary material, equation (B.19), in relative terms, $\varepsilon^{+}$ decreases compared with the limiting strain $\varepsilon_{l}^{+}$. However, $\varepsilon_{l}^{+}$ itself increases with v according to equation (3.4) or equivalently electronic supplementary material, equation (B.16). Hence, even though (due to the decreased $T^{+}$) $\varepsilon^{+}$ is further from $\varepsilon_{l}^{+}$ in relative terms, it still might increase in absolute terms as v increases, which is what we see in figure 4*b* in the various cases with v≤1/2, say.

Indeed, it is only for very small v, such that limiting strains $\varepsilon_{l}^{+}$ are likewise small, that film strains $\varepsilon^{+}$ are always small in absolute terms regardless of the strain imposed $v\tau^{+}$: the solutions of [1] would then apply. However, those solutions cease to apply if v increases. In that case, note also that even if $v\tau^{+}\gg 1$, then the value of $T^{+},\langle T^{\pm }\rangle ,$ which according to equation (3.2) or equivalently electronic supplementary material, equation (B.18), grows only logarithmically, need not be exceedingly large. Thus, again $\varepsilon^{+}$ need not be exceedingly close to $\varepsilon_{l}^{+}$. This is particularly evident in figure 4*b* in the case where v=1/2, which exhibits a slow approach to a limiting $\varepsilon_{l}^{+}$ value, which for this specific velocity turns out to be unity in line with predictions of equation (3.4) or equivalently electronic supplementary material, equation (B.16). The slow fashion in which $\varepsilon^{+}$ approaches unity when v=1/2 is captured by electronic supplementary material, equation (B.20).

Yet other features of figure 4*b* are the cases with v>1/2. These are seen to reach $\varepsilon^{+}=1$ after some finite imposed strain, and surfactant transport then becomes plastic in order to keep $\varepsilon^{+}$ fixed at that value thereafter. This situation is discussed further in the next section.

#### (ii) Conditions for surfactant flux to become plastic

**Figure 5.**
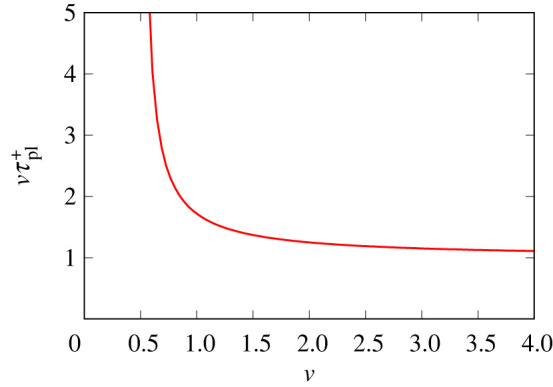
Imposed strain $v\tau_{\mathrm{pl}}^{+}$ versus v. Specifically, $v\tau_{\mathrm{pl}}^{+}$ is the strain that, when imposed on the stretched film, causes $\varepsilon^{+}$ to reach unity, and the surfactant flux onto the film then becomes plastic, transferring thereafter as much surfactant as is needed to keep $\varepsilon^{+}$ fixed. (Online version in colour.)

The imposed strain $v\tau_{\mathrm{pl}}^{+}$ required for the system to become plastic (as given by electronic supplementary material, equation (B.22) or equation (C.7) depending on the v value) is plotted in figure 5 as a function of v in the domain v>1/2. Very large strains need to be imposed if v is only slightly greater than 1/2, but as v increases, the value of $v\tau_{\mathrm{pl}}^{+}$ falls. The smallest possible value of $v\tau_{\mathrm{pl}}^{+}$ is unity and is reached only when v→∞. Having now identified which conditions do not allow the surfactant transport to become plastic, and which conditions do allow this, we can proceed to examine the surfactant content on stretched films.

#### (iii) Surfactant on film during extension

**Figure 6.**
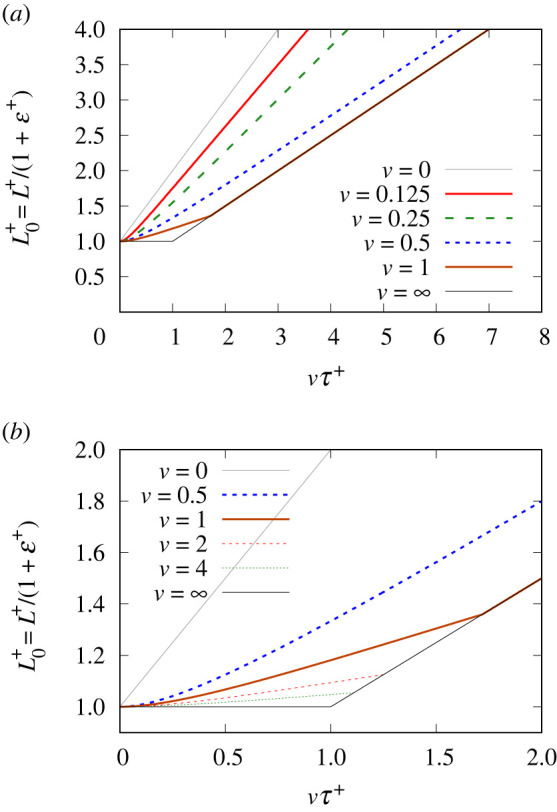
Stretched film: $L_{0}^{+}\equiv L^{+}/(1+\varepsilon^{+})$ versus $v\tau^{+}$. (*a*) Zoomed out view, and (*b*) zoomed in view, considering also some larger values for stretching velocity v. (Online version in colour.)

The value of $L_{0}^{+}\equiv L^{+}/(1+\varepsilon^{+})$ is a measure of the surfactant on the stretched film: specifically, the dimensionless quantity $L_{0}^{+}$ is the amount of surfactant currently on the film relative to the amount on it initially. This is plotted in figure 6 as a function of imposed strain $v\tau^{+}$ for various stretching velocities v. To evaluate this, equation (3.6) (equivalently electronic supplementary material, equation (B.19)) is required, along with electronic supplementary material, equation (B.5). In all cases, in the limit of very small imposed strain, the initial rate of change of $L_{0}^{+}$ vanishes owing to lack of any initial tension difference (see also electronic supplementary material, equation (B.9)). However, moving towards larger imposed strains, we start to see $L_{0}^{+}$ increasing.

There are, however, two generic behaviours. The first behaviour exhibited for v<1/2 (seen clearly in the zoomed out view in figure 6*a*) is that even after a fairly small imposed strain, $L_{0}^{+}$ asymptotes to a straight line behaviour. The value of $L_{0}^{+}$ does not grow quite as fast as that of $L^{+}$ but is close to it. Only for v→0 do we obtain $L_{0}^{+}=L^{+}$. The second behaviour exhibited for v>1/2 (seen clearly in the zoomed in view in figure 6*b*) is that $L_{0}^{+}$ grows slowly with $v\tau^{+}$ at first, until eventually it is only half as large as $L^{+}$. We then see a sudden change in the rate of change of $L_{0}^{+}$, maintaining $L_{0}^{+}=L^{+}/2$ thereafter. This condition is achieved sooner for larger v, but once it is achieved, the value of $L_{0}^{+}$ depends on the imposed strain but not on the individual v. The case where v=1/2 is intermediate between these two aforementioned behaviours: it asymptotes to the line $L_{0}^{+}=L^{+}/2$ but never actually reaches it.

### (c) Incorporating both driving and relaxation phases

The results presented thus far concerned just the driving phase when the motor is switched on. In the present section, we consider both the driving phase and the subsequent relaxation phase. First, we consider compression followed by relaxation (§4ci) and after that, stretch followed by relaxation (§4cii). Some additional discussion on how to compare the compression–relaxation and stretch–relaxation results can be found in electronic supplementary material, appendix D.

#### (i) Compressing a film and then relaxing

**Figure 7.**
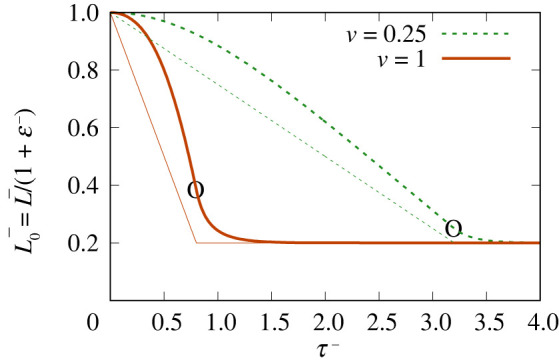
Compressed film: $L_{0}^{-}\equiv L^{-}/(1+\varepsilon^{-})$ versus $\tau^{-}$ both during and after motor motion. The thinner straight lines indicate the corresponding values of $L^{-}$. The circled points represent the motor being switched off. (Online version in colour.)

In figure 7, we show the evolution of $L_{0}^{-}\equiv L^{-}/(1+\varepsilon^{-})$ for a compressed film, with two different compression velocities, v=0.25 and v=1. In the figure, these $L_{0}^{-}$ values (which measure the amount of surfactant on the film) are contrasted with $L^{-}$ (measuring instantaneous film length). To evaluate $L_{0}^{-}$, equation (3.5) (equivalently, electronic supplementary material, equation (B.14)) and electronic supplementary material, equation (B.2), are required. For both v values, the motor is switched off when the film is compressed to 0.2 times its original length. Equation (3.7) or equivalently electronic supplementary material, equation (B.23), now applies, while $L^{-}$ is held fixed.

For both cases where v=0.25 and v=1, initially the rate of change $L_{0}^{-}$ is zero (as noted already in §4aii), but as the motor-driving phase proceeds, the slope of the $L_{0}^{-}$ curve shifts away from zero and (particularly in the case where v=0.25) tends towards a nearly constant slope. When the driving phase ceases, the higher velocity (i.e. v=1) case has managed to retain more surfactant on the film than the v=0.25 one does. In both cases, however, $L_{0}^{-}$ continues to decrease after the motor motion ceases, eventually relaxing back to the same final value as $L^{-}$. Moreover, if we compare the time scale for the driving phase (e.g. the time scale for $L_{0}^{-}$ to reach a constant slope) with the time scale for the relaxation phase, it is clear from figure 7 that the relaxation time scale is shorter. This follows from equation (3.7) (equivalently, electronic supplementary material, equation (B.23)) when $L_{m}^{-}$ (the final $L^{-}$ value when the motor is switched off) is rather smaller than unity, corresponding to a rapid decay of $\varepsilon^{-}$, certainly more rapid than the evolution of $\varepsilon^{-}$ during the motor-driving phase.

#### (ii) Stretching a film and then relaxing

**Figure 8.**
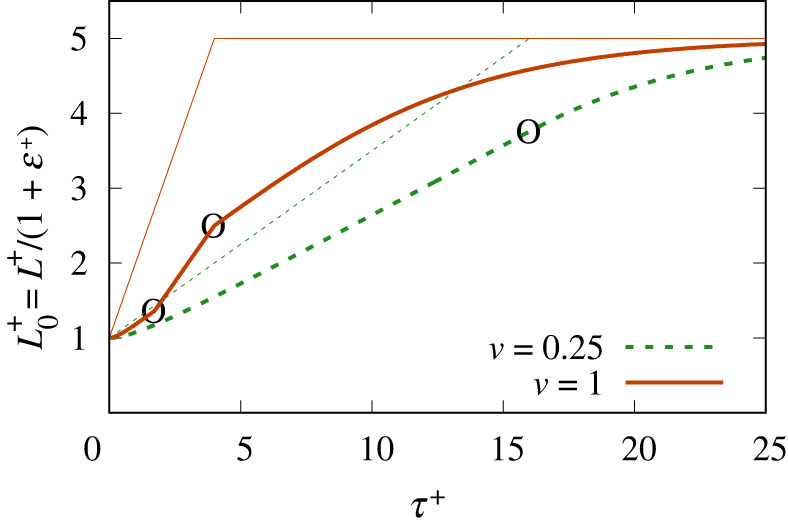
Stretched film: $L_{0}^{+}\equiv L^{+}/(1+\varepsilon^{+})$ versus $\tau^{+}$ both during and after motor motion. The thinner straight lines indicate the corresponding values of $L^{+}$. In the case where v=0.25, the circled point represents the motor being switched off. In the case where v=1, the lower circled point represents the surfactant flux becoming plastic and the upper circled point represents the motor being switched off. (Online version in colour.)

In figure 8, we show $L_{0}^{+}\equiv L^{+}/(1+\varepsilon^{+})$ in a a stretched film case, with the film now being stretched to five times its original length and the motor then being switched off. The velocities considered, v=0.25 and v=1, are the same as those in figure 7.

We consider the v=0.25 case first. Equation (3.6) (equivalently, electronic supplementary material, equations (B.19) and (B.5)) apply up to the point that the motor is switched off. After motor switch off, equation (3.7) or equivalently electronic supplementary material, equation (B.23), applies instead, with $L^{+}$ now held fixed. As already noted in §4biii, at initial time, the rate of change of $L_{0}^{+}$ is zero, but $L_{0}^{+}$ soon evolves (after some characteristic time scale) towards growing at a nearly constant rate. After the motor is switched off, $L_{0}^{+}$ continues to grow albeit by fairly modest amounts, relaxing towards $L_{m}^{+}$ (which is the value of film length $L^{+}$ when the motor is switched off). However, the relaxation time scale is now longer than the characteristic time scale during the motor-driving phase. This is in line with the predictions of equation (3.7) or equivalently electronic supplementary material, equation (B.23), which suggest that the relaxation time in this dimensionless system itself scales like $L_{m}^{+}$. The quantity $L_{m}^{+}$ is now rather larger than unity, implying a long relaxation time and hence slow relaxation.

The case where v=1 is a little different. During the motor-driving phase, the value of $L_{0}^{+}$ does not grow nearly as fast as that of $L^{+}$. Equation (3.6) (equivalently, electronic supplementary material, equations (B.19)) and (B.5)) still apply during this stage, although for v=1, equation (3.6) simplifies to electronic supplementary material, equation (C.8). However, this situation soon ceases to apply. Instead, we see a sudden increase in the rate of change of $L_{0}^{+}$ at the instant when $L_{0}^{+}$ becomes only half of $L^{+}$. However, this increase in the rate of change of $L_{0}^{+}$ is undone when the motor motion stops and the relaxation phase begins. In that case, as has been mentioned, equation (3.7) or equivalently electronic supplementary material, equation (B.23), now applies with $L^{+}$ held fixed, and this predicts $\varepsilon^{+}$ falling. Hence, not only does $L_{0}^{+}\equiv L^{+}/(1+\varepsilon^{+})$ rise, but the ratio $L_{0}^{+}/L^{+}=1/(1+\varepsilon^{+})$, which was formerly equal to one half, must rise also.

We then see quite a long time scale relaxation, after which $L_{0}^{+}$ eventually reaches the same value as $L^{+}$ (i.e. it reaches the value $L_{m}^{+}$). When v=1, the amount of increase in $L_{0}^{+}$ during the relaxation phase is very significant (i.e. very significant amounts of surfactant transport occur even during this phase, which follows from electronic supplementary material, equation (B.6), with $L^{+}$ fixed but $\varepsilon^{+}$ decaying all the way from unity to zero during the relaxation phase). Indeed, when v=1, more surfactant is transported during the relaxation phase than during the motor-driving phase.

A final comment we make is that both figures 7 and 8 are plotted in terms of dimensionless time. These figures indicate that various different dimensionless time scales are involved during compression, relaxation after compression, stretch, and relaxation after stretch. What the various time scales correspond to in dimensional variables is discussed in electronic supplementary material, appendix D.

This now completes our analysis of the model of [1] considering the general case in which the compression or stretch imposed upon films is significant. To summarize, the model admits very different behaviours between low imposed velocity and high imposed velocity, between compression and stretching, and between motor-driving and relaxation phases. Overall conclusions are discussed in the next section.

## 5. Conclusion

We have considered a model (originally proposed by Bussonnière & Cantat [1]) for how surfactant is transported from film to film around a meniscus within a foam. The key idea of the model is that there is a relation (in effect, a constitutive relation) between film-to-film surfactant transport rate and tension differences between adjacent films. Tension differences are in turn related to film strains using a Gibbs elasticity parameter. Although the Gibbs elasticity could in principle depend on strain, it is treated here as constant, which leads to a simple analytically tractable model.

The model has been applied to describe surfactant transport in a five-film device (two compressed films on one side and two stretched films on the other, plus a central film joining them). The model makes a distinction between the strain imposed on the films by a motor that drives them and the actual strain developed on the film elements themselves. By assumption, the central film is not strained, but strains certainly develop in the compressed and stretched films. The work of [1] restricted consideration, at least as far as solutions for the five-film device as a whole were concerned, to situations in which the strain imposed on either the compressed or stretched films, and consequently also the actual strain developed on the film elements, is small. This was done despite the fact that the constitutive model employed could actually cope with large strains. Indeed, if the imposed strain is large enough and if it is also imposed rapidly enough, strain on the film elements need not be small. This then is the case considered in the present work. Despite the fact that strains can now be rather large, the model remains analytically tractable, albeit solutions become more complicated than those presented by Bussonnière & Cantat [1].

Knowing the instantaneous length of each film, and the instantaneous strain on the film elements within it, gives a measure of the instantaneous amount of surfactant contained on each film, and thereby also the amount of surfactant that has been transported off the compressed film and/or onto the stretched one. In particular, in the case of the compressed film, even if it is compressed down to just a tiny fraction of its original length, the strain within the film itself remains limited at a value set by the compression velocity. The larger the compression velocity, the larger the magnitude of the limiting compressive strain. This then determines the amount of surfactant retained by the compressed film, but much of the surfactant originally in place has simply been transported off it.

The case of the stretched film is somewhat different. If the film is stretched slowly enough, the strain on it reaches (as in the compressed case) a limiting value that depends on velocity. This limiting value can be approached if the stretch that is imposed is large, but only if the velocity of stretching is not too large. On the other hand, if the film is stretched fast enough for long enough, it reaches a certain strain at which the behaviour suddenly changes. Surfactant flux jumps to a larger value than before to prevent the film strain (and the film tension that depends upon it) from growing any further. The reason that strain cannot grow further is associated with so-called geometrical frustration: locally, near the meniscus, different sides of a film behave differently, and in particular strong stretching leads to very strong surfactant depletion but only on one side of the film. The strain we see moving slightly away from the meniscus is, however, not quite so large, as the two sides adjust towards an average strain value. The film strain only begins to relax after the imposed stretching is stopped. However, significant surfactant mass transfer onto the film continues even as relaxation of the strain proceeds.

Although the model predicts interesting behaviour, it is worth reflecting that, as formulated here, it has only been studied for a five-film device, not for a more general foam. Nonetheless, the compression and stretching processes that occur in the five-film device capture some of the processes that also occur in a general foam. Specifically, the topological transformations (mentioned in the introduction) that occur very generally in foams involve certain films being compressed and shrinking down until they vanish, while newly created films are strongly stretched after they are formed. Moreover, as a newly created film stretches, any films that neighbour it must shrink to compensate. What is clear is that these topological transformation processes impose large strains on films participating in them. In that respect, the fact that the present work has managed to obtain model solutions involving large imposed strains seems to be particularly significant.

As formulated, though, one of the issues with the model is that it is a lumped parameter model, which allows strains (and film tensions that depend on them) to vary from film to film but not along individual films. Thus, the rate-determining step for surfactant transport is in effect assumed to be transport around the meniscus, rather than transport along the films themselves. The relation proposed between surfactant flux at the meniscus and film-to-film tension difference should apply to a general foam (indeed, there is no reason to suppose it applies only for the five-film device). However, a challenge is to identify whether the tension difference can always be treated as a lumped parameter for an entire film, or whether instead some more local measure of tension difference must be used. Addressing questions like this can then help to elucidate the role that surfactant physical chemistry will have in determining foam rheology.

## Data Availability

All results presented here are obtained from formulae provided in the appendices in electronic supplementary material. Data are provided in electronic supplementary material [61].
